# Feedback GAP: pragmatic, cluster-randomized trial of goal setting and action plans to increase the effectiveness of audit and feedback interventions in primary care

**DOI:** 10.1186/1748-5908-8-142

**Published:** 2013-12-17

**Authors:** Noah M Ivers, Karen Tu, Jacqueline Young, Jill J Francis, Jan Barnsley, Baiju R Shah, Ross E Upshur, Rahim Moineddin, Jeremy M Grimshaw, Merrick Zwarenstein

**Affiliations:** 1 Department of Family and Community Medicine, Women’s College Hospital, 76 Grenville Street, Toronto, ON M5S 1B2, Canada; 2 Institute for Clinical Evaluative Sciences, G1 06, 2075 Bayview Avenue, Toronto, ON M4N 3 M5, Canada; 3 Institute of Health Policy Management and Evaluation, University of Toronto, Health Sciences Building, 155 College Street, Suite 425, Toronto, ON M5T 3 M6, Canada; 4 Department of Family and Community Medicine, University of Toronto, 500 University Avenue 5th Floor, Toronto, ON M5T 1 W7, Canada; 5 University Health Network-Toronto Western Hospital Family Health Team, 399 Bathurst Street, West Wing, 2nd Floor, Toronto, ON M5T 2S8, Canada; 6 School of Health Sciences, City University London, Northampton Square, London EC1V 0HB, UK; 7 Sunnybrook Health Sciences Centre, 2075 Bayview Avenue, Toronto, ON M4N 3 M5, Canada; 8 Dalla Lana School of Public Health, University of Toronto, 155 College Street, 6th floor, Toronto, ON M5T 3 M7, Canada; 9 Bridgepoint Health, 14 St. Matthews Road, Toronto, ON M4M 2B5, Canada; 10 Clinical Epidemiology Program, Ottawa Health Research Institute, 1053 Carling Avenue, Administration Building, Room 2-017, Ottawa, ON K1Y 4E9, Canada; 11 Department of Medicine, University of Ottawa, 501 Smyth Road, Box 206, Ottawa, Ontario, K1H 8 L6, Canada; 12 Department of Family Medicine, Centre for Studies in Family Medicine, Schulich School of Medicine and Dentistry, Western University, 245-100 Collip Circle, Western Research Park, London, Ontario N6G 4X8, Canada

## Abstract

**Background:**

Audit and feedback to physicians is a commonly used quality improvement strategy, but its optimal design is unknown. This trial tested the effects of a theory-informed worksheet to facilitate goal setting and action planning, appended to feedback reports on chronic disease management, compared to feedback reports provided without these worksheets.

**Methods:**

A two-arm pragmatic cluster randomized trial was conducted, with allocation at the level of primary care clinics. Participants were family physicians who contributed data from their electronic medical records. The ‘usual feedback’ arm received feedback every six months for two years regarding the proportion of their patients meeting quality targets for diabetes and/or ischemic heart disease. The intervention arm received these same reports plus a worksheet designed to facilitate goal setting and action plan development in response to the feedback reports. Blood pressure (BP) and low-density lipoprotein cholesterol (LDL) values were compared after two years as the primary outcomes. Process outcomes measured the proportion of guideline-recommended actions (*e.g.*, testing and prescribing) conducted within the appropriate timeframe. Intention-to-treat analysis was performed.

**Results:**

Outcomes were similar across groups at baseline. Final analysis included 20 physicians from seven clinics and 1,832 patients in the intervention arm (15% loss to follow up) and 29 physicians from seven clinics and 2,223 patients in the usual feedback arm (10% loss to follow up). Ten of 20 physicians completed the worksheet at least once during the study. Mean BP was 128/72 in the feedback plus worksheet arm and 128/73 in the feedback alone arm, while LDL was 2.1 and 2.0, respectively. Thus, no significant differences were observed across groups in the primary outcomes, but mean haemoglobin A1c was lower in the feedback plus worksheet arm (7.2% versus 7.4%, p<0.001). Improvements in both arms were noted over time for one-half of the process outcomes.

**Discussion:**

Appending a theory-informed goal setting and action planning worksheet to an externally produced audit and feedback intervention did not lead to improvements in patient outcomes. The results may be explained in part by passive dissemination of the worksheet leading to inadequate engagement with the intervention.

**Trial registration:**

ClinicalTrials.gov NCT00996645

## Background

Audit and feedback is often the foundation of quality improvement (QI) projects aiming to close the gap between ideal and actual practice. Audit and feedback is known to improve quality of care but there is variability in the magnitude of effect observed [1]. This variability may be attributed to the nature of the targeted behavior, the context, and the characteristics of the recipient, as well as to the design of the audit and feedback intervention itself [2]. The Cochrane review of audit and feedback found that feedback is more effective when sent more than once, delivered by a supervisor or senior colleague in both verbal and written formats, and when it includes both explicit targets and an action plan [3]. However, these conclusions are based on indirect comparisons from meta-regressions and are thus less reliable than those that would be generated directly from head-to-head trials of different approaches to providing audit and feedback. Further, there is little information to guide operationalization of these factors [4]. For example, few audit and feedback trials explicitly describe goal setting or action planning as part of the intervention, and those trials that did appeared to deliver this component of the intervention in various ways [5,6]. Although action planning is a familiar activity in clinical practice, the plan can more effectively lead to behavior change if it includes two key elements: an ‘if’ statement (specifying contextual factors that will trigger the action) and a ‘then’ statement (specifying precisely the action to be taken) [7]. In the context of feedback and goals, implementation intention-based action plans could increase goal-directed behaviors possibly by increasing both self-efficacy (confidence in ones ability to perform an action effectively) and goal-commitment (degree to which the person is determined to achieve the goal) [8].

For patients with diabetes, audit and feedback is known to modestly improve processes of care, as well as blood pressure (BP) and glycemic control [9,10]. In this trial, we aimed to build on the extant knowledge regarding audit and feedback by asking not whether it can improve care, but whether it could be modified to be more effective to improve processes of care related to patients with chronic disease, including diabetes and ischemic heart disease (IHD). Our hypothesis was that feedback reports delivered to family physicians regarding care patterns for patients with chronic disease would draw attention to a discrepancy between actual and desired quality of care (*e.g.*, fewer patients than expected with BP recently measured) and that a worksheet accompanying the feedback to facilitate goal-setting and action-planning would increase the likelihood that family physicians would act to improve quality of care (*e.g.*, identify and more aggressively treat patients with BP above target).

## Methods

### Study design

This was a two-arm, pragmatic cluster-trial conducted in primary care. To reduce the risk of contamination, randomization was at the level of the primary care clinic. Each physician in the intervention clinics received feedback accompanied by a goal-setting and action-planning worksheet, while each physician in the clinics allocated to usual care received feedback unaccompanied by the worksheet. Feedback reports addressed guideline-based quality indicators for patients in their practice with diabetes and/or IHD. Such patients are at elevated risk of cardiovascular events, especially if they have a history of both conditions [11], and guidelines recommend similar processes of care as well as control of BP and cholesterol to reduce this risk [12,13]. The trial was pragmatic in that it sought to determine if the intervention could be effective under usual circumstances: the goal-setting and action-planning worksheet was designed to be readily scalable and was delivered with minimal supports; the usual feedback arm was not standardized with respect to co-interventions; patient-level outcomes were assessed from databases with unobtrusive measurement of compliance; and analysis was by intention-to-treat [14]. The protocol has been previously published [15] and is summarized below. This study received approval from the Research Ethics Office at Sunnybrook Health Sciences Centre (271–2006) and registered at ClinicalTrials.gov (NCT00996645).

### Setting

In the province of Ontario, patients with chronic conditions such as diabetes and stable ischemic heart disease are generally managed in primary care, mostly by family physicians. There is no co-pay for doctor visits or laboratory tests for Ontarians, but medications are covered by the provincial drug plan only for the elderly and those on social assistance. The majority of primary care providers in the province work in groups and are paid by a mix of capitation and fee for service.

### Participants and data collection

Participants were family physicians throughout Ontario who signed data-sharing agreements with the Electronic Medical Record Administrative data Linked Database (EMRALD), held at the Institute for Clinical Evaluative Sciences (ICES). EMRALD has developed mechanisms to extract, securely transfer, and de-identify the electronic medical record (EMR) data for analysis at ICES, maintaining strict standards for confidentiality [16]. Family physicians were originally invited to participate in EMRALD through convenience sampling of EMR users and all EMRALD contributors consented to this study. Physicians with less than one year of experience using their EMR or with less than 100 active adult patients enrolled in their practice were excluded. Included patients were over age 18 at the start of the trial, were enrolled with their family physician throughout the study, and had diabetes and/or IHD. Only patients with one or more visits at least one year prior to the trial to were included to ensure that enough data existed in the EMR to assess quality of care and to ensure that providers were not audited for transient or new patients. EMRALD has validated algorithms to identify patients with diabetes and IHD, which do not require special data input by physicians [17,18].

### Allocation

Practices were allocated using minimization (conducted by the study analyst using the free software, MINIM [19]) to achieve balance on baseline values of the primary outcomes and on the number of eligible patients in each cluster [3]. Using the baseline data for each cluster, these variables were classified as high or low using the median value as the cut-point. After recruitment was completed, practices were allocated simultaneously to ensure low risk of bias related to allocation concealment, as per Cochrane Effective Practice and Organization of Care Group criteria [20].

### Intervention

The intervention was developed through an iterative process and piloted with family physicians, as described previously [15]. Each physician received a package (Table 1) by courier every six months for two years featuring feedback reports describing the aggregate percentage of patients with diabetes and/or IHD meeting quality targets, along with explanatory documents and self-reflection surveys to be completed for continuing medical education credits (CME). For each disease condition, the report fit on one page and for every quality target, the aggregate performance achieved by the participating physician was compared to the score achieved by the top 10% of participating physician performers [21]. See Additional file 1 for prototype feedback reports. The frequency was limited to twice yearly for two years due to capacity of the research team, and the reports included only aggregate data (no patient-specific information) due to potential privacy risks of sending patient-specific information.

**Table 1:** Intervention components and description

	
Feedback reports	One page focusing on patients with diabetes and one on patients with heart disease. Aggregate rather than patient-specific data provided. Performance compared to top 10% of peers, presented in tables and bar graphs.
Explanatory document	One page description of how data was generated, including possible limitations.
Suggestions document	One page with clinical recommendations (ie. managing muscle aches for patients taking statins) and generic quality improvement strategies (*i.e.*. work with administrative staff to encourage patients to have periodic visits only for chronic disease management)
Continuing medical education form	Two-page self-reflection survey required by the College of Family Physicians to earn continuing medical education credits related to practice audits.

Physicians randomized to the feedback plus worksheet arm also received a one-page worksheet appended to the feedback along with the standard CME survey. The worksheet was designed to facilitate goal-setting and implementation intention-based action-plans, using the ‘if’ and ‘then’ formulation explained above [8]. See Additional file 2 for prototype of worksheet. Participants were asked to submit the worksheet along with the CME surveys in order to process their CME credits.

Prior to the second cycle of feedback reports, the College of Family Physicians of Canada implemented the use of standardized forms to earn continuing medical education credits for practice audits. Therefore, the CME surveys changed during the trial. The original surveys asked participants what they learned about diabetes and IHD care, intention to change practice, and asked about potential barriers to change. The revised surveys asked participants to ‘make a decision about your practice,’ to declare ‘what will you have to do to integrate these decisions,’ and to continue to reassess the practice change and make further plans to improve. Given the nature of the intervention, blinding of physicians was not possible, but they were not aware of the exact nature of the intervention being tested.

### Outcomes

Outcomes were monitored using validated processes to analyze data collected from participants’ EMRs. There were two patient/disease-level primary outcomes and one professional practice/process-level primary outcome. The patient-level primary outcomes were the patients’ most recent low-density lipoprotein cholesterol (LDL) and systolic BP values, if tested within 24 or 12 months, respectively. The process-level primary outcome was a composite process score indicating whether patients received prescriptions and tests in accordance with relevant guidelines [22-27]. Patients received a composite process score with a maximum of six, as outlined in Table 2 which we multiplied by 100 to report the score as a percentage.

**Table 2:** Composite process score calculated for each patient as primary process outcome

**Quality indicator (for each patient receives a score)**	**Diabetes (maximum score = 6)**	**IHD (maximum score = 6)**	**Both diabetes + IHD (multiply by 6/7 for max score = 6)**
BP test in 6 M	X	X	X
A1C test in 6 M	X		X
FBG test in 24 M		X	
LDL test in 12 M	X	X	X
ACR test in 12 M	X		X
Rx ASA		X	X
Rx Statin	X	X	X
Rx ACE/ARB	X	X	X

*IHD* ischemic heart disease, *BP* blood pressure, *A1C* haemoglobin A1c, *FBG* fasting blood glucose, *LDL* low density lipoprotein cholesterol, *ACR* urinary albumin-to-creatinine ratio, *ASA* aspirin, *ACE/ARB* angiotensin-modifying agent.

Secondary outcomes were chosen because they were thought to reflect the targets of action by family physicians receiving the feedback. Each of the items in the composite process score was assessed, plus glycemic control (haemoglobin A1c level, HbA1c), the proportion meeting targets recommended in guidelines for LDL (<2 mmol/L), and BP (<130/80 mmHg in diabetes and <140/90 mmHg in IHD), and prescriptions rates for insulin and beta blockers. While not every patient requires each prescription or investigation, we anticipated balance between groups in the proportion of eligible patients. Therefore, increases in aggregate proportions of processes performed indicate general intensification of treatment for patients with these conditions.

### Analysis

Primary analysis was performed on an intention-to-treat basis, using patient level variables, combining patients with diabetes and/or IHD. Because the intervention was directed to the physician, final analysis was limited to patients with diabetes and/or IHD who were enrolled in their physician’s practice throughout the trial. We used linear mixed models (SAS MIXED procedure) for continuous variables to estimate the mean difference in each outcome between arms, together with their 95% confidence interval (CI). For dichotomous variables, we used generalized estimating equation models (SAS GENMOD procedure) and estimated relative risk and the 95% confidence interval using log-binomial regression [28]. We used generalized linear models to examine random variation for each outcome at both physician and practice levels (SAS GLIMMIX procedure). The clustering of patients within physicians was accounted for using random effect models and if the practice-level was also significant we included both levels when assessing outcomes. We ran a model for each outcome with and without adjustment for baseline values of the dependent variable. The adjustment for baseline values of the dependent variable was carried out by specifying the pre-intervention measure of the outcome as a covariate. We planned this approach a priori because although there were thousands of patients, we were not confident that allocation of only 14 clinics would result in adequate balance at baseline [3,29].

Planned sub-group analyses were performed on patients with only IHD, only diabetes, or both, to assess the same outcome variables. We hypothesized that patients with only IHD would have lower quality of care scores at baseline and greater potential for improvement during the intervention because locally it has received relatively less attention with respect to QI initiatives and because identifying patients in the EMR with IHD is more difficult than identifying patients with DM due to the lack of relevant laboratory results or specific medications. A planned per-protocol analysis assessed whether full completion of the intervention worksheet resulted in improved outcomes in general and for the specific clinical topics in participants’ goal statements. All analyses were carried out using the SAS Version 9.2 statistical program (SAS Institute, Cary, NC, USA).

The number of participating practices and eligible physicians providing data to EMRALD determined the sample size. With 54 physicians from 14 practices initially providing data and consenting to this trial and a presumed intra-cluster correlation of 0.05, we estimated 80% power to find an absolute difference in LDL of 0.32 mmol/L and in systolic BP of 7 mmHg, based on pilot data [15].

## Results

**Figure 1:**
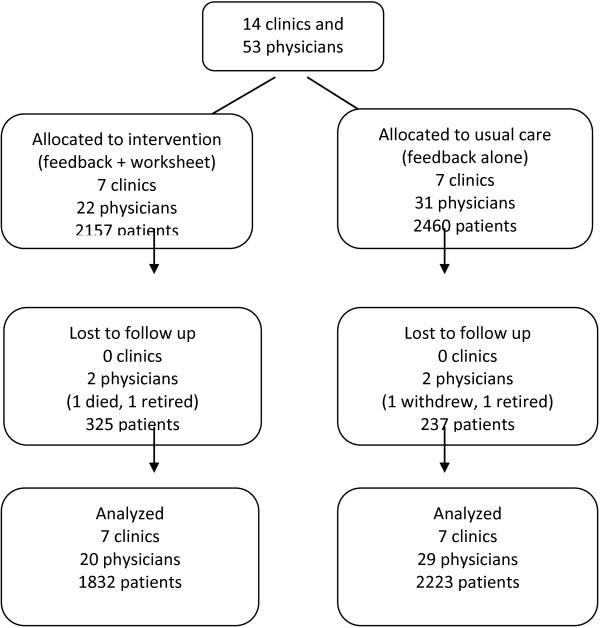
Cluster-Patient flow diagram.

Cluster and patient flow are described in Figure 1. Just prior to allocation, one physician stopped practicing. Thus, the study began with 4,617 patients cared for by 53 physicians from 14 clinics. At baseline (August 2010), 22 physicians cared for 2,157 patients with diabetes and/or IHD in the feedback plus worksheet arm and 31 physicians cared for 2,460 patients were in the usual feedback arm. During the two-year trial two physicians from each arm were lost to follow up. Of the 562 patients lost to follow up, 175 belonged to these four physicians, and 166 changed physicians during the study. Average age of patients lost to follow up was 68 years and 44% were female, reflecting the underlying distribution of those allocated.

### Comparability at baseline

Cluster and participant characteristics are described in Table 3 and Table 4, respectively. The median number of included patients per clinic was 234, (interquartile range (IQR) 186 – 508) and the median per physician was 81 (IQR, 46 – 111). One-half of the practices (7/14, 50%) were located in urban settings. About one-half of the physicians were female (25/53, 47%). Physicians had been in practice for a median of 19 years (IQR, 9 – 24) and had been using their EMR for a median of 7 years (IQR 6 – 7). Intervention physicians were more likely to be male, with more years experience, and located in rural settings. They also tended to have smaller practices overall but with more eligible patients. Patients averaged 65.6 years and 44.4% were female. Most included patients (3,435, 74.4%) had diabetes, the remainder had IHD (25.6%); one-eighth (12.4%) had both diabetes and IHD. The mean weight of included patients was 89 kilograms (standard deviation [SD] = 26, 501 missing). Mean BP was 130/74 (systolic SD = 17, diastolic SD = 11, 467 missing), with 55.1% achieving target, and mean LDL was 2.31 (SD = 0.9, 674 missing), with 67.7% achieving target. The mean process composite score was 72 (SD = 26). Values for these variables and other process measures were similar across groups, except for greater proportion of patients in the feedback plus worksheet arm group than the feedback alone arm with a recent BP test (85% versus 74%) and HbA1c test (79% versus 69%).

**Table 3:** Baseline characteristics of clinics, and family physicians

	**Feedback plus worksheet**	**Feedback alone**
**Clinic characteristics (N = 14)**	7	7
Total patients (median, IQR)	3,121 (2,258 – 2,671)	4,432 (3,488 – 8,201)
Eligible* patients (median, IQR)	205 (189 – 389)	257 (211 – 499)
Location (n, % rural)	4 (57%)	2 (29%)
**Physician characteristics (N = 53)**	22	31
Roster size (median, IQR)	1,010 (596 – 1,416)	1,291 (884 – 1,696)
Sex (n, % female)	6 (27%)	19 (61%)
Years in practice (median, IQR)	23 (16 – 25)	15 (7 – 22)
Years using EMR (median, IQR)	7 (6.7 – 7.9)	6 (6.2 – 6.6)
Eligible* patients (median, IQR)	77 (49 – 120)	179 (62 – 216)

^*^Eligible patients are those meeting inclusion criteria with diabetes and/or heart disease.

IQR = interquartile range, % = proportion.

**Table 4:** Baseline characteristics of patients

	**Feedback plus worksheet**	**Feedback alone**	**ICC**
**Patients (total = 4,617)**	2,157	2,460	-
IHD only	545 (25%)	637 (26%)	-
DM only	1,329 (62%)	1,534 (62%)	-
IHD and DM	283 (13%)	289 (12%)	-
Age (mean years ± sd)	67 ±14	65 ±14	-
Female	906 (42%)	1,251 (46%)	-
WT (mean kg ± sd)	88 ±22	90 ±29	0.047
Systolic BP (mean mmHg ± sd)	130 ±18	129 ±17	0.058
Diastolic BP (mean mmHg ± sd)	73 ±11	75 ±11	0.079
LDL (mean mmol/l ± sd)	2.3 ±0.9	2.3 ±0.9	0.053
HbA1c^ (mean% ± sd)	7.4 ±1.4	7.3 ±1.4	0.011
LDL at target~	665 (31%)	826 (34%)	0.051
BP at target~	1,019 (47%)	1,054 (43%)	0.071
BP test in 6 M	1,839 (85%)	1,831 (74%)	0.183
A1C test in 6 M^	1,267 (79%)	1,250 (69%)	0.079
FBG test in 24 M	1,904 (88%)	2,134 (87%)	0.091
LDL test in 12 M	1,574 (73%)	1,770 (72%)	0.096
ACR test in 12 M^	1,044 (65%)	1,232 (68%)	0.195
Rx ASA*	602 (73%)	657 (71%)	0.092
Rx Statin	1,463 (68%)	1,757 (71%)	0.051
Rx ACE/ARB^	1,153 (71%)	1,243 (68%)	0.068
Rx Beta blocker*	497 (60%)	562 (61%)	0.008
Rx insulin^	339 (21%)	391 (21%)	0.006
Composite process score (mean ± sd)	73 ±25	71 ±27	0.106

Legend: ICC = Intra-cluster correlation reported at physician-level, ^ = Calculated for patients with diabetes, * Calculated for patients with IHD, ~ = Target BP 130/80 for diabetes but 140/90 for IHD and Target LDL < 2, *IHD* = ischemic heart disease, *BP* = blood pressure, *A1C* = haemoglobin A1c, *FBG* = fasting blood glucose*, LDL* = low density lipoprotein cholesterol*, ACR* = urinary albumin-to-creatinine ratio, *ASA* = aspirin, *ACE/ARB* = angiotensin-modifying agent, *kg* = kilogram, *sd* = standard deviation.

### Intention-to-treat analyses

There were 4055 patients cared for by 49 physicians from 14 clinics in the final analysis. The primary and secondary outcomes at the end of the trial are summarized in Table 5. No clinically or statistically significant differences were observed across groups in the primary outcomes in both the adjusted and unadjusted models. Mean systolic BP was 128 in both arms (adjusted mean difference = -0.05; 95% CI -2.11, 2.02) and diastolic BP was 72 in the feedback plus worksheet arm and 73 in the feedback alone arm (adjusted mean difference = -0.72; 95% CI -2.18, 0.75). LDL was 2.1 in the feedback plus worksheet arm and 2.0 in the feedback alone arm (adjusted mean difference = 0.04; 95% CI -0.02, 0.10). The mean composite score was 72 in the feedback plus worksheet arm and 70 in the feedback alone arm [adjusted mean difference = 1.76; 95% CI -1.4, 4.9]. Mean HbA1c in patients with diabetes was lower in the feedback plus worksheet arm (7.2% versus 7.4%; adjusted mean difference -0.2; 95% CI -0.3, -0.1). A greater proportion of patients in the feedback plus worksheet arm had their BP tested within six months (81% versus 69%, adjusted relative risk [aRR] = 1.2; 95% CI 1.1, 1.3) and more achieved target BP (53% versus 46%, aRR 1.2; 95% CI 1.0 – 1.3). No other outcomes were significantly different across the arms.

**Table 5:** Outcomes for patients receiving feedback plus the goal-setting and action-planning worksheet versus feedback alone

	**Feedback + worksheet**	**Feedback alone**	**Model-based differences in quality of care outcomes**					
	**N = 1,832**	**N = 2,223**						
**Continuous outcomes**			**MD**	**95% CI**	***Missing***	**Adjusted MD**	**95% CI**	***Missing***
Systolic BP	128 ±18	128 ±16	0.35	-2.24, 2.95	*554*	-0.05	-2.11, 2.02	*743*
Diastolic BP	72 ±11	73 ±11	-1.48	-3.34, 0.38	*554*	-0.72	-2.18, 0.75	*743*
LDL	2.1 ±0.8	2.0 ±0.8	0.04	-0.06, 0.14	*643*	0.04	-0.02, 0.10	*927*
HbA1c%^	7.2 ±1.3	7.4 ±1.5	-0.24	-0.38, -0.09	*572*	-0.20	-0.33, -0.06	*794*
Composite score	72 ±26	70 ±28	4.85	-0.52, 10.21	*0*	1.76	-1.37, 4.89	*0*
**Dichotomous outcomes**			**RR**	**95% CI**		**Adjusted RR**	**95% CI**	
LDL at target	866 (47%)	1,119 (50%)	0.97	0.85, 1.13		0.97	0.90, 1.06	
BP at target	965 (53%)	1,023 (46%)	1.21	1.05, 1.40		1.15	1.02, 1.29	
BP test in 6 M	1,491 (81%)	1,541 (69%)	1.25	1.11, 1.42		1.16	1.06, 1.26	
A1C test in 6 M^	947 (71%)	1,067 (65%)	1.12	0.98, 1.28		1.07	0.95, 1.21	
FBG test in 24 M	1,596 (87%)	1,918 (86%)	1.03	0.97, 1.10		1.03	0.98, 1.08	
LDL test in 12 M	1,217 (66%)	1,480 (67%)	1.03	0.93, 1.16		1.02	0.93, 1.12	
ACR test in 12 M^	851 (64%)	1,060 (65%)	1.02	0.87, 1.20		1.05	0.93, 1.20	
Rx ASA*	552 (75%)	615 (73%)	1.03	0.95, 1.11		0.99	0.96, 1.03	
Rx statin	1,309 (72%)	1,686 (76%)	0.96	0.89, 1.03		0.97	0.94, 1.00	
Rx ACE/ARB^	647 (83%)	679 (84%)	1.05	0.97, 1.14		0.98	0.95, 1.00	
Rx beta blocker*	440 (59%)	514 (61%)	1.00	0.90, 1.10		0.99	0.95, 1.03	
Rx insulin^	351 (26%)	426 (26%)	0.99	0.85, 1.16		1.01	0.99, 1.04	

All models adjusted for clustering. Adjusted models also controlled for baseline values of dependent variable.

Legend: ^Analysis restricted to patients with diabetes, *Analysis restricted to patients with IHD, ~ Target BP 130/80 for diabetes and 140/90 for IHD and Target LDL<2.

*MD* mean difference, *RR* relative risk, *IHD* ischemic heart disease, *BP* blood pressure, *A1C* haemoglobin A1c, *FBG* fasting blood glucose, *LDL* low density lipoprotein cholesterol, *ACR* albumin-to-creatinine ratio, *ASA* aspirin, *ACE/ARB* angiotensin-modifying agent, *Rx* active prescription, *CI* confidence interval, *M* months.

After adjusting for variation at the level of the physicians, there was no further significant variation at the level of the clinic so that all models included a random variable only for the physician. Patients with no recent values for BP or LDL did not differ across arms with respect to sex, or proportion with diabetes and/or IHD. Those with missing values for BP were also similar with respect to age, but patients with missing values for LDL from the feedback plus worksheet arm tended to be older than those in the feedback alone arm (68 years versus 63 years; p = 0.001).

LDL values and diastolic BP decreased slightly in both study arms over time, while systolic BP decreased only in the feedback plus worksheet arm (Additional file 3: Table S6). HbA1c also decreased in the feedback plus worksheet arm, but increased slightly in the feedback only arm. The proportion of patients with BP and LDL at target increased in both arms during the study period. The proportion with an LDL test within 12 months and the proportion with a statin prescribed also improved over time in both arms, but the proportion with BP measured within 6 months decreased in both arms and the proportion prescribed an anti-hypertensive (beta blocker or angiotensin-modifying agent) did not change over time except for a small increase in angiotensin-modifying agents in the feedback alone arm. Insulin prescribing increased in both arms over time, but testing of HbA1c and fasting blood glucose decreased.

Results of the planned sub-group analyses for patients with only diabetes, only IHD, or both conditions are described in Additional file 3: Table S7, Table S8, Table S9. The results indicate that patients with IHD are treated more aggressively than patients with only diabetes and that patients with both conditions are treated most aggressively. Mean BP and LDL values were lowest and the composite process score was highest among patients with diabetes and IHD.

### Per-protocol analyses

One-half of physicians in the intervention arm completed the worksheet (10/22, 45%); of those, one-half responded only once. Restricting analysis for the primary outcomes to the 10 physicians who completed the intervention worksheet indicated non-statistically significant improvement in the feedback plus worksheet arm for systolic BP (126 versus 128, adjusted mean difference -0.8; 95% CI -3.1, 1.5) and for the composite score (73 versus 70, adjusted mean difference 2.6; 95% CI -2.0, 7.2), but a slight decrease in LDL (2.1 versus 2.0, adjusted mean difference 0.1; 95% CI 0.0, 0.2). The most common goals set by participants in the worksheet for their patients with diabetes were for achievement of target BP (*i.e.*, increase the proportion of patients meeting BP target, three times) and increasing testing rates of urinary albumin-to-creatinine ratio (three times). The most common goals for IHD were for achievement of target LDL (three times) and increasing prescription rates of ASA (five times). Patients belonging to the 10 participants who completed the worksheets more often achieved BP targets but the effect was not statistically significant (52% versus 46%, aRR = 1.11; 95% CI 0.97, 1.27). Testing for urinary albumin was similar between arms (66% versus 65%, aRR = 1.00; 95% CI 0.86, 1.17), as was achievement of LDL targets (48% versus 50%, aRR = 0.99; 95% CI 0.90, 1.09). Prescribing rates of ASA was higher among participants who completed the worksheets, but the model-based difference was not statistically significant, as baseline rates of ASA prescribing were also higher in this group (67% versus 57%, aRR = 0.99; 95% CI 0.95, 1.04).

## Discussion

We found no difference in the primary outcomes of BP and cholesterol levels and no difference in the composite process score when providers were given feedback plus a goal-setting and action-planning worksheet compared to feedback alone. While it was a secondary outcome and should be interpreted cautiously, the intervention did result in improved glycemic control to a similar extent as many other complex QI strategies for diabetes [30]. We also observed that BP and cholesterol improved in both arms, as well as one-half of the process outcomes, which emphasizes the importance of controlled studies when testing strategies aiming to improve quality of care.

It remains plausible that completing a goal-setting and action-planning exercise could enhance the effectiveness of feedback [1]. Unfortunately, only 10 out of 22 physicians in the intervention arm completed the intervention worksheet and only five of these completed it more than once. Poor compliance (with minimal supports) limited our ability to test the effects of action planning in this pragmatic trial. One-half of the goals set by active participants were behavioral (what will I do) and one-half were outcome-oriented (what would I like to happen as the result of what I do). For actions plans to be most effective, they must very specifically relate to behavioral goals, not outcome goals [31]. Thus, it would seem that one-half of participants who completed the worksheet did so ineffectively, and there is a need to explore how to make action-planning activities more salient and usable. More active, practice-based supports may be needed to implement the development of goals and action plans. For instance, one randomized control trial found that feedback reports plus structured peer interactions in which goals and action plans for improvement were discussed was more effective than feedback alone [32]. A recent Canadian cross-sectional study showed that data management support for patient identification and recall plus assistance by allied health providers with standardized testing and prescribing was associated with improved quality in primary care [33].

To result in behavior change, those receiving feedback must be dissatisfied with their performance, meet a threshold level of self-efficacy for improvement, and be committed to the goal [7,34]. In our separately reported embedded qualitative evaluation [35], we found that self-efficacy was low, as many practices lacked the necessary QI infrastructure to take action. For example, no one was responsible for searching the EMR to identify patients who may require reassessment. Our qualitative work also found that participants were not highly committed to achieving the targets described in the feedback reports. There was uncertainty regarding the impact on patient outcomes of achieving targets perceived to be aggressive. Many expressed concern that practice-level QI efforts would be at odds with their attempts to achieve patient-centered care. It would appear that although Canadian family physicians generally agree with and accept guideline-based best-practice targets for diabetes and IHD [36], achievement of best-practice targets for chronic disease management was not perceived as urgent compared to other tasks, especially given the relatively high quality of care already achieved. Yet, even if mean performance was acceptable, many patients stand to benefit from improved processes of care. In such settings, to increase goal commitment it may be necessary to first address limited self-efficacy by providing more active supports for QI [37], as there is evidence that self-efficacy influences goal commitment [34]. Recognizing that feedback alone is sometimes not enough to change provider behaviors, further improvements have been sought by pairing feedback with intensive co-interventions, such as academic detailing [38] or practice facilitation [39]. In the Cochrane review, pairing educational outreach with audit and feedback was found to increase desired professional behavior [3]. However, these intensive interventions are costly and more cost-efficient approaches may exist.

The particular nature of the feedback intervention used in this study may have played an important role in the poor uptake of the intervention worksheet. Qualitative work conducted in the Veterans Affairs health system in the USA also indicates that high performing healthcare organizations tend to deliver feedback with more actionable information [40]. It is possible that the participants did not feel that achieving higher scores on their feedback was achievable because only aggregate data was provided [41]. It is also possible that concerns regarding data validity allowed participants to resolve any cognitive dissonance arising from the results provided in the feedback without needing to commit resources to improvement [42]. For instance, the explanatory notes accompanying the reports described that if relevant tests were conducted by specialists but were not received into the EMR in a standardized format they would not be included in the feedback. In addition, the presence of multiple competing priorities is known to mitigate achievement of particular guideline recommendations [43], and this may be highly appropriate for patients with significant symptoms from concomitant illness (*e.g.*, severe depression, cancer) or reduced life expectancy. The Cochrane review indicated that feedback was less effective when targeting many indicators reflecting chronic disease management than when targeting a specific behavior, and feedback intervention theory suggests that feedback should direct attention to a specific task in order to most reliably change behavior [44]. It has also been observed that feedback may be more effective when participants choose standards [5], and when presented by senior colleagues [3], whereas externally generated feedback focusing on a multitude of guideline-based best practices were used in this study. Therefore, the feedback may have been more salient—and the intervention worksheet may have had greater impact—if it focused on quality indicators chosen by participants to be high priority, and delivered by carefully selected opinion leaders [45] with clear and readily achievable tasks for improved scores.

Some limitations in this study warrant further discussion. The lack of a pure control group limits our ability to comment on the impact of this particular audit and feedback intervention on quality of care. However, our approach was necessary because participants expected something in return for contributing data. Furthermore, feedback is known to work for these outcomes [10] such that our interest was in determining whether a simple enhancement could increase feedback effectiveness. Although we used minimization to achieve baseline balance successfully for the primary outcomes, differences in cluster-level characteristics remained. In addition, the study analyst had access to the allocation list; this non-blinding could theoretically create bias, but the same validated algorithms were used to assess outcomes for each study group. We also acknowledge the potential for measurement bias as investigations were counted only if results were available in the EMR and tests performed by specialists may be missed. For outcomes related to investigations and treatments, data in EMRALD compare well with (and often out-perform) administrative databases [46]. While the trial should balance reasons for misclassification or missing data, we observed that those without a recent LDL test tended to be older in the intervention arm. We took a pragmatic approach to intervention delivery, limiting the number of reminders and supports to mimic expected conditions if the intervention were to be widely implemented. This may explain the limited completion rate of the worksheet and raises a question about the role of pragmatic health services trials when evaluating ‘new’ interventions. In this case, more data regarding how to support implementation would have been useful prior to embarking on a trial with this type of design. We did embed a qualitative evaluation to explore this, but participants focused on the usefulness of the particular audit and feedback intervention used in this trial rather than the goal-setting and action-planning worksheets [35]. It is also important to note that the secondary, per-protocol analyses are at risk of bias in favour of the intervention.

In addition, a number of factors may have limited our ability in this study to find differences between intervention arms. First, while this study did include thousands of patients, they were clustered within only 14 clinics and the intra-cluster correlations for disease-level primary outcomes were larger than expected from pilot data. Second, physicians voluntarily provide data to EMRALD and many participating clinics were involved in other QI interventions. Thus, these clinics may be more innovative and may also be achieving a higher level of evidence-based care than most other primary care providers, potentially decreasing both generalizability and the likelihood of finding an effect in this study. Furthermore, we observed that the composite process score was highest among patients with both diabetes and IHD indicating that participating providers appropriately intensified monitoring and management in patients at greatest risk and suggesting the possibility that further gains may be limited by a ceiling effect. Third, risk for type 2 error is exacerbated in trials comparing similar interventions (*i.e.*, head-to-head trials) where anticipated effect sizes are not expected to be large. Finally, differences between groups may also have been more difficult to identify if goal-setting and action-planning aspects of the intervention were duplicated by the revised CME surveys mandated by the College of Family Physicians. During the trial, 52% of physicians (14/27) in the usual feedback group and 54% (12/22) in the feedback plus worksheet arm completed and returned at least one CME survey. Unlike the first iteration of CME surveys, which asked participants ‘what have you learned,’ the revised surveys explicitly asked participants to make a decision about their practice and to identify how to integrate the decision into practice. Though less specific or directive than the intervention worksheets, these questions may have similarly prompted participants to set goals and develop action plans.

In conclusion, we found no effect of adding a theory-informed goal-setting and action-planning worksheet to an audit and feedback intervention. Unfortunately, passive dissemination of this worksheet led to inadequate engagement with the intervention. In the context of primary care practices with minimal QI infrastructure, CME credit alone may not be enough incentive to encourage engagement in goal-setting or action planning activities. To maximize the impact of audit and feedback and to ensure that QI in primary care is prioritized, relevant stakeholders, including professional colleges, associations, and health system payers, should consider the need for further supports to carry out practice-based QI.

## Competing interests

The authors declare that they have no competing interests.

## Authors’ contributions

NI, JG, KT, and MZ conceived the idea. NI, JY, and RM conducted the analyses. NI prepared the manuscript. All authors have made substantial contributions to the research design, have edited the manuscript critically, read and have approved of the final version.

## Supplementary Material

Additional file 1 **Feedback Intervention.** Prototype of feedback report that all participants received. Click here for file

Additional file 2 **Goal-setting and Action-plan Worksheet for Intervention Arm.** Prototype of the intervention tested in the trial. Click here for file

Additional file 3 **Supplementary Tables.** Change from baseline (Table S6) and sub-group analyses (Tables S7, S8, S9). Click here for file
