# Supplementary material for: Feedback GAP: pragmatic, cluster-randomized trial of goal setting and action plans to increase the effectiveness of audit and feedback interventions in primary care
Source: Implement Sci. 2013 Dec 17;8:142. doi: 10.1186/1748-5908-8-142 (PMC3878579; doi:10.1186/1748-5908-8-142)
Supplement: Additional file 1 — Feedback Intervention. Prototype of feedback report that all participants received. [file 1748-5908-8-142-S1.docx]

| 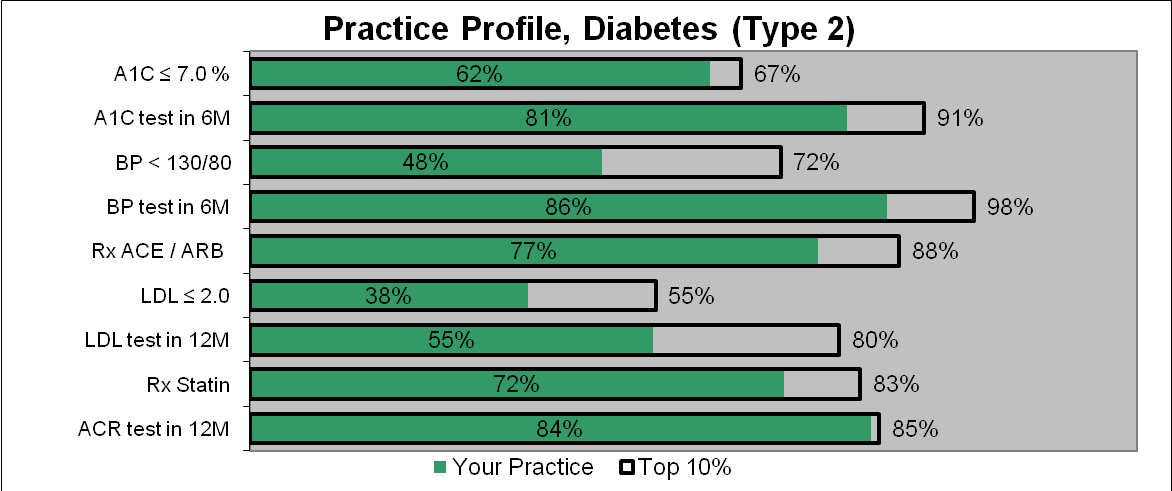 | **PHYSICIAN ID#:** | | | x | |  | |  | |  | |  | | | | |  |  | |  |  |
| --- | --- | --- | --- | --- | --- | --- | --- | --- | --- | --- | --- | --- | --- | --- | --- | --- | --- | --- | --- | --- | --- |
|  | Approximately 12% of your rostered adult patients have diabetes, and 30% of these patients also have ischemic heart disease | | | | | | | | | | | | | | | |  |  | |  |  |
|  | Overall in this study, 7% of rostered adult patients have diabetes, and 19% of these patients also have ischemic heart disease | | | | | | | | | | | | | | | |  |  | |  |  |
|  | *Your* diabetic patients are 68 years old on average and are 57% male. All diabetic patients in the study average 63 years and are 55% male. | | | | | | | | | | | | | | | | |  | |  |  |
|  |  | |  | | |  | |  | |  | |  | |  | | | |  | |  |  |
|  | **Targets** | | **Your Practice** | | | Top 10% | |  | |  | |  | |  | | | |  | |  |  |
|  | A1C ≤ 7.0 % | | **62%** | | | 67% | |  | | "Top 10%" = the score achieved by 10% of physicians | | | | | | | | | |  |  |
|  | A1C test in 6M | | **81%** | | | 91% | |  | | with the best score for each target. | | | | | | | | | |  |  |
|  | BP < 130/80 | | **48%** | | | 72% | |  | |  | |  | |  | | | |  | |  |  |
|  | BP test in 6M | | **86%** | | | 98% | |  | | *(This data is based on your most recent EMR data upload, May,2010)* | | | | | | | | | |  |  |
|  | Rx ACE / ARB | | **77%** | | | 88% | |  | |  | |  | | | | |  |  | |  |  |
|  | LDL ≤ 2.0 | | **38%** | | | 55% | |  | |  | |  | | | | |  |  | |  |  |
|  | LDL test in 12M | | **55%** | | | 80% | |  | |  | |  | | | | |  |  | |  |  |
|  | Rx Statin | | **72%** | | | 83% | |  | |  | |  | | | | |  |  | |  |  |
|  | ACR test in 12M | | **84%** | | | 85% | |  | | ACR = urinary albumin creatinine ratio (microalbumin) | | | | | | |  |  | |  |  |
| \| 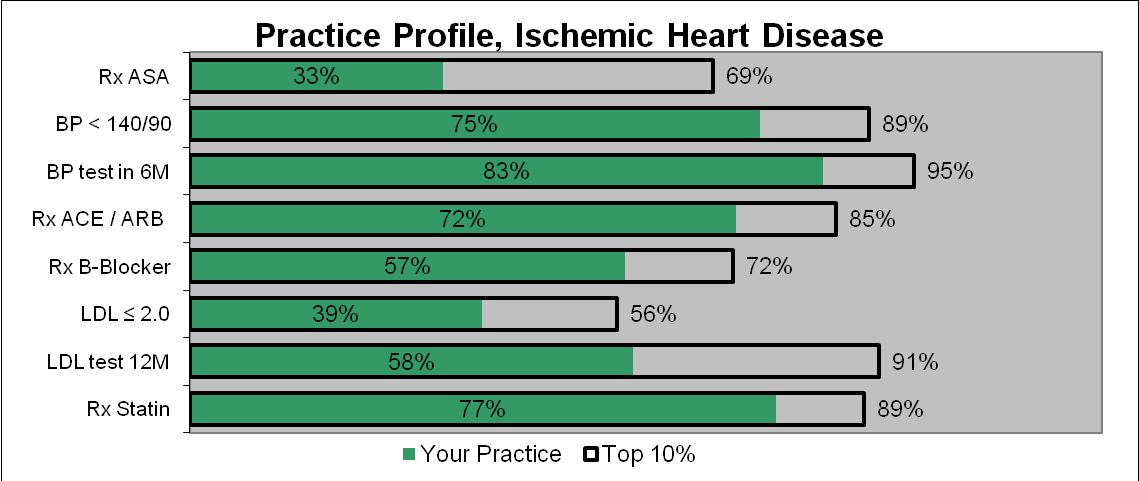 \| \| --- \| | |  | | |  | |  | |  | |  | |  | | |  | | |  | |  |
|  | |  | | |  | |  | |  | |  | |  | | |  | | |  | |  |
|  | |  | | |  | |  | |  | |  | |  | | |  | | |  | |  |
|  | |  | | |  | |  | |  | |  | |  | | |  | | |  | |  |
|  | |  | | |  | |  | |  | |  | |  | | |  | | |  | |  |
|  | |  | | |  | |  | |  | |  | |  | | |  | | |  | |  |
|  | |  | | |  | |  | |  | |  | |  | | |  | | |  | |  |
|  | |  | | |  | |  | |  | |  | |  | | |  | | |  | |  |
|  | |  | | |  | |  | |  | |  | |  | | |  | | |  | |  |
|  | |  | | |  | |  | |  | |  | |  | | |  | | |  | |  |
|  | |  | | |  | |  | |  | |  | |  | | |  | | |  | |  |
|  | |  | | |  | |  | |  | |  | |  | | |  | | |  | |  |
|  | |  | | |  | |  | |  | |  | |  | | |  | | |  | |  |
|  | |  | | |  | |  | |  | |  | |  | | |  | | |  | |  |
|  | |  | | |  | |  | |  | |  | |  | | |  | | |  | |  |
|  | |  | | |  | |  | |  | |  | |  | | |  | | |  | |  |
|  | |  | | |  | |  | |  | |  | |  | | |  | | |  | |  |
|  | |  | | |  | |  | |  | |  | |  | | |  | | |  | |  |
|  | |  | | |  | |  | |  | |  | |  | | |  | | |  | |  |
|  | | **PHYSICIAN ID#:** | | | x | |  | |  | |  | |  | | |  | | |  | |  |
|  | | Approximately 9% of your rostered adult patients have ischemic heart disease, and 41% of these patients also have diabetes | | | | | | | | | | | | | |  | | |  | |  |
|  | | Overall in this study, 5% of rostered adult patients have ischemic heart disease, and 28% of these patients also have diabetes | | | | | | | | | | | | | |  | | |  | |  |
|  | | *Your* IHD patients are 71 years old on average and are 74% male. All IHD patients in the study average 70 years and are 65% male. | | | | | | | | | | | | | | | | |  | |  |
|  | |  |  | | | |  | |  | |  | |  | |  | | | |  | |  |
|  | | **Targets** | **Your Practice** | | | | Top 10% | |  | |  | |  | |  | | | |  | |  |
|  | | Rx ASA | **33%** | | | | 69% | |  | | "Top 10%" = the score achieved by 10% of physicians | | | | | | | | | |  |
|  | | BP < 140/90 | **75%** | | | | 89% | |  | | with the best score for each target. | | | | | | | | | |  |
|  | | BP test in 6M | **83%** | | | | 95% | |  | |  | |  | |  | | | |  | |  |
|  | | Rx ACE / ARB | **72%** | | | | 85% | |  | | *(This data is based on your most recent EMR data upload, May,2010)* | | | | | | | | | |  |
|  | | Rx B-Blocker | **57%** | | | | 72% | |  | |  | |  | |  | | | |  | |  |
|  | | LDL ≤ 2.0 | **39%** | | | | 56% | |  | |  | | | | | | | | | |  |
|  | | LDL test 12M | **58%** | | | | 91% | |  | |  | |  | |  | | | |  | |  |
|  | | Rx Statin | **77%** | | | | 89% | |  | |  | |  | |  | | | |  | |  |
