# Supplementary material for: Feedback GAP: pragmatic, cluster-randomized trial of goal setting and action plans to increase the effectiveness of audit and feedback interventions in primary care
Source: Implement Sci. 2013 Dec 17;8:142. doi: 10.1186/1748-5908-8-142 (PMC3878579; doi:10.1186/1748-5908-8-142)
Supplement: Additional file 3 — Supplementary Tables. Change from baseline (Table S6) and sub-group analyses (Tables S7, S8, S9). [file 1748-5908-8-142-S3.docx]

**Supplementary Tables**

**Table S6. Change in outcomes over time by intervention arm, adjusted for effects of clustering**

|  | **Feedback +Worksheet** | | | **Feedback Alone** | | |
| --- | --- | --- | --- | --- | --- | --- |
| **Continuous outcomes** | **Mean difference** | **95% CI** | *Missing* | **Mean difference** | **95% CI** | *Missing* |
| Systolic BP | -1.83 | -0.68, -2.98 | *257* | -0.96 | -1.99, 0.07 | *708* |
| Diastolic BP | -1.30 | -0.62, -1.98 | *257* | -1.74 | -1.08, -2.41 | *708* |
| LDL | -0.24 | -0.19, -0.30 | *549* | -0.26 | -0.32, -0.21 | *647* |
| HbA1c%^ | -0.14 | -0.03, -0.25 | *374* | 0.12 | 0.02, 0.22 | *604* |
| Composite score | -3.09 | -1.49, -4.69 |  | -1.81 | -3.31, -0.31 |  |
| **Dichotomous outcomes** | **Relative Risk** | **95% CI** |  | **Relative Risk** | **95% CI** |  |
| LDL at target | 1.33 | 1.22, 1.44 |  | 1.32 | 1.23, 1.43 |  |
| BP at target | 1.08 | 1.01, 1.16 |  | 1.08 | 1.01, 1.15 |  |
| BP test in 6M | 0.95 | 0.92, 0.98 |  | 0.93 | 0.88, 0.99 |  |
| A1C test in 6M^ | 0.88 | 0.80, 0.97 |  | 0.94 | 0.88, 1.00 |  |
| FBG test in 24M | 0.98 | 0.96, 1.00 |  | 0.99 | 0.97, 1.01 |  |
| LDL test in 12M | 1.12 | 1.06, 1.18 |  | 1.10 | 1.04, 1.16 |  |
| ACR test in 12M^ | 0.92 | 0.88, 0.97 |  | 0.95 | 0.88, 1.01 |  |
| Rx ASA* | 1.00 | 0.97, 1.03 |  | 1.03 | 0.99, 1.09 |  |
| Rx Statin | 1.02 | 1.00, 1.04 |  | 1.06 | 1.03, 1.09 |  |
| Rx ACE/ARB^ | 1.01 | 0.98, 1.04 |  | 1.03 | 1.01, 1.06 |  |
| Rx Beta blocker* | 0.99 | 0.97, 1.02 |  | 0.99 | 0.96, 1.03 |  |
| Rx Insulin^ | 1.24 | 1.16, 1.33 |  | 1.22 | 1.17, 1.28 |  |

Legend: ^Analysis restricted to patients with diabetes, *Analysis restricted to patients with IHD, ~ Target BP 130/80 for diabetes and 140/90 for IHD and Target LDL<2

IHD=ischemic heart disease, BP=blood pressure, A1C=haemoglobin A1c, FBG=fasting blood glucose, LDL=low density lipoprotein cholesterol, ACR=albumin-to-creatinine ratio, ASA=aspirin, ACE/ARB=angiotensin-modifying agent, Rx=active prescription, CI=confidence interval, M=months

**Table S7. Outcomes for patients with diabetes but not IHD, adjusted for effects of clustering**

|  | **Feedback +Worksheet** | **Feedback Alone** | **Model-based differences in quality of care outcomes** | | | | | |
| --- | --- | --- | --- | --- | --- | --- | --- | --- |
|  | **n=1049** | **n=1329** |  |  |  |  |  |  |
| **Continuous outcomes** |  |  | **MD** | **95% CI** | Missing | **Adjusted MD** | **95% CI** | Missing |
| Systolic BP | 129 ±17 | 129 ±16 | 0.55 | -2.19, 3.28 | 358 | 0.00 | -2.23, 2.21 | 479 |
| Diastolic BP | 73 ±10 | 75 ±11 | -1.49 | -3.28, 0.30 | 358 | 0.83 | -2.31, 0.65 | 479 |
| LDL | 2.2 ±0.9 | 2.1 ±0.8 | 0.07 | -0.05, 0.18 | 643 | 0.05 | -0.02, 0.13 | 521 |
| HbA1c | 7.1 ±1.3 | 7.4 ±1.5 | -0.29 | -1.06, 0.48 | 470 | -0.32 | -1.00, 0.36 | 658 |
| Composite score | 69 ±28 | 67 ±30 | 1.73 | -1.13, 4.61 | 0 | 0.81 | -2.84, 4.46 | 0 |
| **Dichotomous outcomes** |  |  | **RR** | **95% CI** |  | **Adjusted RR** | **95% CI** |  |
| LDL at target | 456 (43%) | 622 (47%) | 0.95 | 0.81, 1.11 |  | 0.95 | 0.86, 1.04 |  |
| BP at target | 397 (38%) | 434 (33%) | 1.20 | 0.99, 1.46 |  | 1.18 | 0.99, 1.41 |  |
| BP test in 6M | 838 (80%) | 916 (69%) | 1.21 | 1.08, 1.37 |  | 1.12 | 1.03, 1.23 |  |
| A1C test in 6M | 742 (71%) | 856 (64%) | 1.11 | 0.97, 1.27 |  | 1.07 | 0.94, 1.21 |  |
| FBG test in 24M | 932 (89%) | 1169 (88%) | 1.03 | 0.97, 1.09 |  | 1.02 | 0.97, 1.07 |  |
| LDL test in 12M | 704 (67%) | 903 (68%) | 1.01 | 0.90, 1.15 |  | 1.00 | 0.90, 1.10 |  |
| ACR test in 12M | 650 (62%) | 839 (63%) | 1.02 | 0.87, 1.21 |  | 1.06 | 0.94, 1.21 |  |
| Rx ASA | 599 (57%) | 620 (47%) | 1.28 | 1.07, 1.52 |  | 1.01 | 0.97, 1.06 |  |
| Rx Statin | 683 (65%) | 918 (69%) | 0.94 | 0.86, 1.03 |  | 0.97 | 0.94, 1.00 |  |
| Rx ACE/ARB | 727 (69%) | 898 (68%) | 1.05 | 0.95, 1.14 |  | 0.97 | 0.94, 1.00 |  |
| Rx Insulin | 251 (24%) | 333 (25%) | 0.94 | 0.78, 1.13 |  | 1.00 | 0.98, 1.04 |  |

Adjusted models controlled for baseline values of dependent variable.

Legend: ^ = Analysis restricted to patients with diabetes, *Analysis restricted to patients with IHD, ~ = Target BP 130/80 for diabetes and 140/90 for IHD and Target LDL<2

Acronyms: RR = relative risk, IHD = ischemic heart disease, BP = blood pressure, A1C = haemoglobin A1c, FBG = fasting blood glucose, LDL = low density lipoprotein cholesterol, ACR = albumin-to-creatinine ratio, ASA = aspirin, ACE/ARB = angiotensin-modifying agent, Rx = active prescription, CI = confidence interval, M = months

**Table S8. Outcomes for patients with IHD but not diabetes, adjusted for effects of clustering**

|  | **Feedback +Worksheet** | **Feedback Alone** | **Model-based differences in quality of care outcomes** | | | | | |
| --- | --- | --- | --- | --- | --- | --- | --- | --- |
|  | **n=467** | **n=560** |  |  |  |  |  |  |
| **Continuous outcomes** |  |  | **MD** | **95% CI** | Missing | **Adjusted MD** | **95% CI** | Missing |
| Systolic BP | 127 ±19 | 126 ±16 | 0.93 | -2.60, 4.48 | 148 | 0.94 | -2.10, 3.99 | 203 |
| Diastolic BP | 71 ±11 | 71 ±11 | -0.38 | -3.09, 2.33 | 148 | 0.20 | -1.99, 2.38 | 203 |
| LDL | 2.0 ±0.7 | 2.0 ±0.8 | 0.16 | -0.11, 0.15 | 643 | 0.05 | -0.02, 0.13 | 521 |
| Composite score | 73 ±25 | 71 ±24 | 1.48 | -1.03, 3.99 | 0 | 2.34 | -0.44, 5.12 | 0 |
| **Dichotomous outcomes** |  |  | **RR** | **95% CI** |  | **Adjusted RR** | **95% CI** |  |
| LDL at target | 218 (47%) | 276 (49%) | 0.98 | 0.78, 1.25 |  | 1.01 | 0.88, 1.16 |  |
| BP at target | 330 (71%) | 362 (65%) | 1.13 | 1.00, 1.27 |  | 1.08 | 0.97, 1.19 |  |
| BP test in 6M | 372 (80%) | 364 (65%) | 1.35 | 1.14, 1.59 |  | 1.26 | 1.11, 1.42 |  |
| FBG test in 24M | 284 (61%) | 322 (57%) | 1.05 | 0.93, 1.18 |  | 1.05 | 0.96, 1.14 |  |
| LDL test in 12M | 99 (21%) | 149 (27%) | 1.11 | 0.95, 1.31 |  | 1.11 | 0.98, 1.26 |  |
| Rx ASA | 366 (78%) | 472 (84%) | 1.00 | 0.91, 1.11 |  | 0.98 | 0.94, 1.02 |  |
| Rx Statin | 329 (70%) | 399 (71%) | 0.95 | 0.88, 1.03 |  | 0.96 | 0.93, 1.00 |  |
| Rx Beta blocker | 249 (53%) | 332 (59%) | 0.93 | 0.81, 1.05 |  | 0.98 | 0.92, 1.05 |  |
| Rx ACE/ARB | 218 (47%) | 276 (49%) | 1.00 | 0.91, 1.11 |  | 1.02 | 0.98, 1.06 |  |

Adjusted models also controlled for baseline values of dependent variable.

Legend: ^Analysis restricted to patients with diabetes, *Analysis restricted to patients with IHD, ~ Target BP 130/80 for diabetes and 140/90 for IHD and Target LDL<2

MD=mean difference, RR=relative risk, IHD=ischemic heart disease, BP=blood pressure, A1C=haemoglobin A1c, FBG=fasting blood glucose, LDL=low density lipoprotein cholesterol, ACR=albumin-to-creatinine ratio, ASA=aspirin, ACE/ARB=angiotensin-modifying agent, Rx=active prescription, CI=confidence interval, M=months

**Table S9. Outcomes for patients with both DM and IHD, adjusted for effects of clustering**

|  | **Feedback +Worksheet** | **Feedback Alone** | **Model-based differences in quality of care outcomes** | | | | | |
| --- | --- | --- | --- | --- | --- | --- | --- | --- |
|  | **n=249** | **n=255** |  |  |  |  |  |  |
| **Continuous outcomes** |  |  | **MD** | **95% CI** | Missing | **Adjusted MD** | **95% CI** | Missing |
| Systolic BP | 127 ±19 | 129 ±18 | -1.66 | -6.53, 3.21 | 43 | -1.54 | -5.52, 2.44 | 49 |
| Diastolic BP | 69 ±11 | 71 ±10 | -2.06 | -5.07, 0.95 | 43 | -0.86 | -3.44, 1.72 | 49 |
| LDL | 2.1 ±0.8 | 2.0 ±0.8 | 0.08 | -0.09, 0.26 | 43 | 0.05 | -0.09, 0.18 | 76 |
| HbA1c | 7.3 ±1.3 | 7.2 ±1.3 | 0.07 | -0.21, 0.36 | 80 | 0.13 | -0.33, 0.19 | 108 |
| Composite score | 79 ±20 | 77 ±23 | 2.97 | -1.08, 7.03 | 0 | 0.24 | -4.00, 4.47 | 0 |
| **Dichotomous outcomes** |  |  | **RR** | **95% CI** |  | **Adjusted RR** | **95% CI** |  |
| LDL at target | 153 (61%) | 167 (65%) | 0.95 | 0.80, 1.14 |  | 0.96 | 0.85, 1.08 |  |
| BP at target | 184 (74%) | 169 (66%) | 1.13 | 0.96, 1.34 |  | 1.13 | 0.99, 1.28 |  |
| BP test in 6M | 222 (89%) | 196 (79%) | 1.22 | 1.04, 1.44 |  | 1.10 | 0.98, 1.24 |  |
| A1C test in 6M | 180 (72%) | 175 (69%) | 1.15 | 0.94, 1.42 |  | 1.10 | 0.93, 1.30 |  |
| FBG test in 24M | 232 (93%) | 235 (92%) | 1.02 | 0.95, 1.10 |  | 1.01 | 0.95, 1.09 |  |
| LDL test in 12M | 184 (74%) | 191 (75%) | 0.98 | 0.85, 1.14 |  | 0.98 | 0.87, 1.10 |  |
| ACR test in 12M | 179 (72%) | 185 (73%) | 1.06 | 0.89, 1.27 |  | 1.03 | 0.88, 1.20 |  |
| Rx ASA | 198 (80%) | 187 (73%) | 1.08 | 0.97, 1.21 |  | 1.02 | 0.98, 1.07 |  |
| Rx Statin | 203 (82%) | 223 (87%) | 0.94 | 0.84, 1.04 |  | 0.98 | 0.92, 1.04 |  |
| Rx ACE/ARB | 209 (84%) | 215 (84%) | 1.00 | 0.92, 1.08 |  | 0.98 | 0.94, 1.03 |  |
| Rx Beta blocker | 172 (69%) | 161 (63%) | 1.10 | 0.96, 1.27 |  | 1.00 | 0.93, 1.07 |  |
| Rx Insulin | 91 (37%) | 77 (30%) | 1.22 | 1.02, 1.47 |  | 1.05 | 1.00, 1.10 |  |

All models adjusted for clustering. Adjusted models also controlled for baseline values of dependent variable.

Legend: ^Analysis restricted to patients with diabetes, *Analysis restricted to patients with IHD, ~ Target BP 130/80 for diabetes and 140/90 for IHD and Target LDL<2

MD=mean difference, RR=relative risk, IHD=ischemic heart disease, BP=blood pressure, A1C=haemoglobin A1c, FBG=fasting blood glucose, LDL=low density lipoprotein cholesterol, ACR=albumin-to-creatinine ratio, ASA=aspirin, ACE/ARB=angiotensin-modifying agent, Rx=active prescription, CI=confidence interval, M=months
